# Development and Validation of a Subjective Health Status Scale for Collegiate Athletes

**DOI:** 10.3390/sports14080326

**Published:** 2026-08-03

**Authors:** Wakako Tatsuta, Mizuho Adachi, Takayo Inayama, Yoshitsugu Fujishima, Mutsumi Ogawa

**Affiliations:** 1 College of Sports Sciences, Nihon University, 3-34-1 Shimouma, Setagaya-ku, Tokyo 154-8513, Japan; 2 Graduate Program in Sciences and Culture, Showa Women’s University, 1-7-57 Taishido, Setagaya-ku, Tokyo 154-8533, Japan; 3 Faculty of Sport Science, Nippon Sport Science University, 1221-1 Kamoshida-cho, Aoba-ku, Yokohama 227-0033, Kanagawa, Japan; 4 Department of Food and Health Sciences, Faculty of Health and Human Development, The University of Nagano, 8-49-7 Miwa, Nagano 380-8525, Japan; 5 Department of Social Psychology, Faculty of Sociology, Toyo University, 5-28-20 Hakusan, Bunkyo-ku, Tokyo 112-8606, Japan

**Keywords:** collegiate athletes, subjective health status, scale development, reliability, validity

## Abstract

Background: Accurate assessment of athletes’ health status is essential for effective nutritional education and condition management. However, existing quality of life instruments do not adequately capture sport-specific health issues experienced by athletes. This study aimed to develop a subjective health status scale for collegiate athletes and examine its reliability and validity. Methods: A cross-sectional survey was conducted among 432 collegiate athletes from two private universities in Tokyo, Japan. Six health-related items concerning gastrointestinal, skin, fatigue, and recovery conditions were developed based on semi-structured interviews with athletes and coaches. Exploratory factor analysis was performed to determine the factor structure, followed by assessments of internal consistency and associations with training satisfaction. Results: Two factors were identified: “Gastrointestinal/Skin condition” and “Fatigue Recovery/Reduction”. Both subscales demonstrated acceptable internal consistency (Cronbach’s α = 0.76 for each) and were positively correlated with training satisfaction (r = 0.44–0.55, *p* < 0.001). Multiple regression analysis showed that both factors significantly predicted training satisfaction (adjusted R^2^ = 0.35, *p* < 0.001), with “Fatigue Recovery/Reduction” showing a stronger predictive effect (β = 0.45). Conclusions: The newly developed scale demonstrated satisfactory reliability and validity and may provide a practical, non-invasive tool for monitoring athlete health and evaluating nutritional education and condition management programs.

## 1. Introduction

In recent years, the importance of nutritional education for athletes has been emphasized internationally [1]. The ultimate goal of nutritional education is not merely to improve knowledge or promote behavioral changes but to achieve the outcome objectives of maintaining and improving health status and quality of life (QOL) [2,3]. To achieve these goals, nutritional education should be implemented systematically as part of comprehensive nutrition management [1].

However, previous nutritional education interventions for athletes have not consistently established or reported outcome goals, educational content, or evaluation methods [4]. One reason is the difficulty of evaluating health status as an outcome indicator. Although simple methods are available for assessing some aspects of physical condition, comprehensive objective assessments, such as blood biomarkers and detailed body composition measurements, are often limited in university athletic settings because of invasiveness, cost, and operational burden. To address these constraints, there is a need for the development of a simple, reliable, and valid subjective assessment index that can measure athletes’ health status. Subjective assessments are non-invasive, can be measured repeatedly, and are suitable for group assessments and continuous monitoring. Indeed, internationally validated QOL instruments such as the 36-Item Short Form Health Survey (SF-36) [5] and the World Health Organization Quality of Life (WHOQOL) [6] allow for a cross-sectional evaluation of physical, psychological, social, and environmental dimensions. However, these QOL instruments have limitations, as they do not sufficiently reflect the specific physical symptoms and health challenges associated with high-frequency, high-intensity athletic training. In fact, most previous nutritional education interventions have focused on outcomes related to dietary behavior and knowledge improvement, while few have directly assessed health status. Thus, the development of a validated subjective indicator that can evaluate changes in health status following educational interventions is an urgent priority.

Verification of validity is essential in scale development. Athletes can only sustain high-quality training when their health status is well maintained, which ultimately contributes to performance enhancement [7]. Therefore, the quality of health directly influences daily training experiences. Previous studies have shown that subjective indices such as mood, perceived stress, and fatigue fluctuate with changes in training load [8]. Hence, when developing a new health status scale for athletes, it is crucial to examine not only self-reported symptoms but also the association between health status and training experience and satisfaction. Training satisfaction was selected as a theoretically relevant external criterion because athletes’ perceived health status is closely related to their daily training experiences and engagement in training. This study focused on this point and examined the validity of the newly developed Subjective Health Status Scale using training satisfaction as a theoretically relevant external criterion. Because no validated scale suitable for the present context was available, a Training Satisfaction Scale was also developed for this study.

Based on the above, the present study aimed to develop a Subjective Health Status Scale that reflects the unique health challenges of collegiate athletes and to examine its reliability and validity.

## 2. Materials and Methods

### 2.1. Participants and Procedures

A cross-sectional survey was conducted between October and December 2024 among collegiate athletes enrolled at two private universities in Tokyo, Japan. Participants were athletes belonging to varsity teams competing in the Division I regional university league or achieving top placements in national-level competitions. To avoid bias in sport type, participants were selected from the five sport categories defined by the International Olympic Committee’s Nutrition for Athletes classification [1]: strength, power, endurance, aesthetic/weight-class, and team sports.

The purpose and outline of the study were first explained to the coaches or managers of each team, and their approval was obtained before data collection. The survey was conducted during the general or specific preparation phase, avoiding competition and off-season periods. A group-administered, paper-based survey was conducted. Before participation, the investigator orally explained the study purpose, survey method, protection of personal information, voluntary participation, and confidentiality. Participants who provided informed consent completed the questionnaire, resulting in 432 valid responses. This study was conducted in accordance with the Declaration of Helsinki and the Ethical Guidelines for Life Science and Medical Research Involving Human Subjects (Japan). The study protocol was approved by the Ethics Committee of the College of Sports Sciences, Nihon University (approval No. 2024-007) and the Ethics Committee of Showa Women’s University (approval no. 24-50).

### 2.2. Measures

The questionnaire assessed subjective health status, training satisfaction, dietary habits (nutrient intake, food intake, dietary behaviors, and predisposing and environmental factors), and demographic characteristics. The analyses in this study used the following items.

#### 2.2.1. Subjective Health Status

Items were developed based on semi-structured interviews conducted with collegiate athletes and coaches [9]. The instructions stated: “Please answer the following questions about your usual health status. Select one option for each item that best describes your condition over the past two weeks.” The six items were: “I feel that I have recovered energy from the previous day in preparation for training.” “I can complete the training without running out of energy.” “I do not experience gastrointestinal discomfort, diarrhea, or constipation.” “My bowel movements are regular.” “My skin condition and complexion are good.” “I rarely catch infections.” Each item was rated on a five-point Likert scale: 5 = “Strongly agree,” 4 = “Somewhat agree,” 3 = “Neutral,” 2 = “Somewhat disagree,” 1 = “Strongly disagree.”

#### 2.2.2. Training Satisfaction

Similarly, items assessing training satisfaction were developed based on the same semi-structured interviews [9]. Participants were instructed: “Please answer the following questions about your usual training situation. Select one option for each item that best describes your condition over the past two weeks.” The seven items were: “I can complete all the planned training for the day.” “I exert my full strength.” “I can concentrate during training.” “I can train without anxiety.” “I can engage positively in training.” “I feel refreshed (pleasant).” “I feel enjoyment during training.” Each item was rated using the same five-point Likert scale described above.

#### 2.2.3. Demographic and Physical Characteristics

Participants reported their sex, living arrangements, sport type, height, and body weight. Body mass index (BMI) was calculated as weight (kg)/height (m)^2^.

### 2.3. Statistical Analysis

Data from the 432 respondents were analyzed in the following steps to confirm the reliability and validity of each scale. Analyses were conducted for both the Subjective Health Status Scale and the Training Satisfaction Scale, as follows: Exploratory factor analysis (EFA), Internal consistency, and Inter-scale relationships. EFA was conducted using the maximum likelihood method with promax rotation, adopting eigenvalues greater than 1.0 as the criterion for factor extraction. Items with factor loadings below 0.40 or with cross-loadings above 0.40 on multiple factors were excluded. Internal consistency was examined using Cronbach’s alpha coefficients. For each factor, the scores of the relevant items were summed to create subscale scores. Pearson’s correlation coefficients were calculated to examine associations among scales. Then, multiple regression analysis was performed to examine the influence of subjective health status on training satisfaction, using the total training satisfaction score as the dependent variable and the two subjective health subscale scores as independent variables (forced entry method). The normality of the variables was assessed before the analyses. Although some variables deviated from normality, parametric analyses were considered appropriate given the relatively large sample size and the reported robustness of parametric methods to moderate violations of normality [10]. All analyses were conducted using IBM SPSS Statistics 29 (IBM Corp., Armonk, NY, USA) with a two-tailed significance level of 5%.

## 3. Results

### 3.1. Participants

A total of 432 collegiate athletes (311 men, 119 women, and 2 others) were included in the analysis. The mean age (SD) was 19.7 (1.1) years. Participant characteristics, including sex, age, and sport categories, are presented in Table 1. Sampling adequacy for factor extraction was confirmed using the Kaiser–Meyer–Olkin (KMO) measure, which indicated appropriate values for both scales (0.74 for the subjective health status scale and 0.87 for the training satisfaction scale). Sport categories were as follows: strength (track and field jump/sprint; *n* = 51), power (track cycling, swimming, rowing, alpine skiing; *n* = 115), endurance (cross-country skiing, Nordic combined, road cycling; *n* = 30), aesthetic/weight-class (boxing, wrestling; *n* = 24), team (soccer, soft tennis, etc.; *n* = 172), and others (*n* = 40). Table 1 also presents the distribution of responses for each item of the subjective health status and training satisfaction scales.

### 3.2. Exploratory Factor Analysis

#### 3.2.1. Subjective Health Status Scale

EFA of the six health-related items yielded eigenvalues of 2.87, 1.04, 0.76, and 0.62, indicating that a two-factor solution was appropriate. Factor 1 consisted of the following items (factor loadings in parentheses): “Bowel movements are regular” (0.86), “I do not experience gastrointestinal discomfort, diarrhea, or constipation” (0.78), and “My skin condition and complexion are good” (0.45). Factor 2 included: “I feel that I have recovered energy from the previous day in preparation for training” (0.85), “I can complete the training without running out of energy” (0.73), and “I rarely catch infections” (0.34). Because the loading for “I rarely catch infections” was below 0.40 (0.34), this item was excluded. EFA was then repeated with the remaining five items, and no additional items were removed. Factor 1 was labeled “Gastrointestinal/Skin condition” and Factor 2 was labeled “Fatigue Recovery/Reduction.” The correlation between the two factors was r = 0.37 (Table 2a).

#### 3.2.2. Training Satisfaction Scale

EFA of the seven items on training satisfaction yielded eigenvalues of 4.25, 0.94, and 0.53, supporting a one-factor solution. Factor loadings were as follows: “I feel refreshed (pleasant)” (0.86), “I feel enjoyment during training” (0.79), “I can engage positively in training” (0.78), “I can concentrate during training” (0.71), “I can train without anxiety” (0.69), “I exert my full strength” (0.69), and “I can complete all the planned training for the day” (0.58) (Table 2b).

### 3.3. Internal Consistency

Cronbach’s alpha coefficients demonstrated adequate internal consistency: 0.76 for Gastrointestinal/Skin condition, 0.76 for Fatigue Recovery/Reduction, and 0.89 for Training Satisfaction (Table 2a,b).

### 3.4. Distribution of Scale Scores

Descriptive statistics and group comparisons for the Subjective Health Status Scale and Training Satisfaction Scale are shown in Table 3. The mean (SD) scores were as follows: Gastrointestinal/Skin condition, 12.6 (2.7; 3 items; range, 3–15); Fatigue Recovery/Reduction, 7.7 (1.8; 2 items; range, 2–10); and Training Satisfaction, 30.5 (4.6; 7 items; range, 7–35).

Group differences by sex and sport category were examined using independent *t*-tests and one-way ANOVA. For Gastrointestinal/Skin condition, men scored significantly higher (mean = 13.0 ± 2.3) than women and others (mean = 11.7 ± 3.2) (t = 4.59, df = 430, *p* < 0.001). No main effect of sport category was observed (F(5, 426) = 0.48, ns). For Fatigue Recovery/Reduction, neither sex (t = 1.40, df = 430, ns) nor sport category (F(5, 426) = 2.00, ns) showed significant differences. For Training satisfaction, men (mean = 30.8 ± 4.5) scored significantly higher than women and others (mean = 29.6 ± 4.8) (t = 2.46, df = 430, *p* < 0.01). No main effect of sport category was observed (F(5, 426) = 2.12, ns).

### 3.5. Validity

Pearson’s correlation coefficients and multiple regression results are shown in Table 4 and Table 5. Significant positive correlations were found between: Gastrointestinal/Skin condition and Fatigue Recovery/Reduction (r = 0.43, *p* < 0.001); Gastrointestinal/Skin condition and Training satisfaction (r = 0.44, *p* < 0.001); and Fatigue Recovery/Reduction and Training satisfaction (r = 0.55, *p* < 0.001). Multiple regression analysis, with training satisfaction as the dependent variable and the two health subscales as predictors, revealed a significant model (R^2^ = 0.36, adjusted R^2^ = 0.35, F(2, 429) = 118.14, *p* < 0.001). Standardized regression coefficients were β = 0.24 (t = 5.64, *p* < 0.001) for Gastrointestinal/Skin condition and β = 0.45 (t = 10.46, *p* < 0.001) for Fatigue Recovery/Reduction, indicating a stronger contribution of the latter.

## 4. Discussion

This study developed a Subjective Health Status Scale reflecting the unique health issues of collegiate athletes and examined its reliability and validity. EFA identified a two-factor structure—“Gastrointestinal/Skin condition” and “Fatigue Recovery/Reduction”—both showing satisfactory internal consistency. Both factors were significantly associated with training satisfaction, with Fatigue Recovery/Reduction exerting a stronger predictive effect. These results indicate that the scale can comprehensively assess the multidimensional health status of collegiate athletes and can serve as a practical outcome indicator in nutritional education and condition management.

The Gastrointestinal/Skin condition factor integrated symptoms related to the gastrointestinal tract and skin. High-intensity and prolonged exercise can induce gastrointestinal symptoms through mechanisms such as redistribution of blood flow and mechanical stress, potentially impairing nutrient absorption and performance [11,12,13]. In addition, skin condition is influenced not only by external factors (e.g., ultraviolet exposure, perspiration) [14,15,16] but also by internal factors such as nutritional status and fatigue accumulation [2,17,18,19]. Furthermore, growing evidence suggests interactions between gut microbiota and skin barrier function or inflammation—the so-called gut–skin axis [10,20,21]. These mechanisms may partly explain why gastrointestinal and skin-related symptoms were grouped into a single factor in the present study. Because athletes can easily recognize such physical sensations, this factor allows for non-invasive, practical monitoring of health conditions without objective testing. Consequently, this factor may serve as a useful subjective indicator for assessing nutritional status and physical condition among collegiate athletes.

In contrast, the Fatigue Recovery/Reduction factor aligns with international sports nutrition guidelines, which emphasize the importance of carbohydrate intake and recovery strategies [1,22,23,24]. Perceived recovery from fatigue reflects the adequacy of energy replenishment and relates to motivation and adherence to training. Therefore, the strong association between this factor and training satisfaction suggests that effective nutritional education may enhance athletes’ perceived recovery, leading to greater satisfaction with training.

In the present study, Gastrointestinal/Skin condition scores were significantly lower in women than in men. The observed sex differences may be partly explained by menstrual cycle–related symptoms and iron deficiency, which are relatively common among female athletes [25]. However, because these factors were not assessed in the present study, the mechanisms underlying the observed sex differences remain unclear and warrant further investigation. The infection-related item, “I rarely catch infections,” was excluded due to its low factor loading. Although susceptibility to infection is an important health indicator, it is strongly influenced by external factors such as seasonal prevalence and living environments [26], which may have hindered stable factor formation. Future studies should reconsider its inclusion while accounting for environmental and seasonal variations.

The newly developed scale comprises only five items and is low-cost and non-invasive, making it highly practical for team-wide screening and daily monitoring. Declines in scores can be detected early, allowing individualized follow-up such as counseling or tailored nutrition guidance. By applying this scale, practitioners can implement an integrated process of goal setting, educational intervention, evaluation, and improvement in nutrition management, thereby promoting systematic health support for athletes.

Several limitations should be noted. First, although this study assumed that better health status may contribute to higher training satisfaction, causality was not directly examined. Future intervention and longitudinal studies are needed to assess the responsiveness of the scale. Second, participants were limited to collegiate athletes from two private universities in Tokyo, and generalization to other populations differing in competition level or cultural background requires further investigation. Third, as the scale relies on self-report, it may be affected by social desirability and mood-related bias [27], which cannot be completely ruled out. Future research should examine associations between subjective scores and objective indices such as blood biomarkers to improve measurement accuracy. Finally, the Fatigue Recovery/Reduction subscale consisted of only two items. Although acceptable reliability was demonstrated, future studies should consider adding items that more appropriately capture the Fatigue Recovery/Reduction construct, thereby enhancing the comprehensiveness of the scale for assessing athletes’ subjective health status.

## 5. Conclusions

This study developed a new Subjective Health Status Scale reflecting the specific health challenges of collegiate athletes and confirmed its reliability and validity. The scale comprises two factors—Gastrointestinal/Skin condition and Fatigue Recovery/Reduction—both significantly associated with training satisfaction. This enables simple and continuous assessment of athletes’ health status in field settings, providing a practical outcome indicator for nutrition education and condition management. Future studies should further evaluate its applicability through educational interventions and comparisons with objective health measures.

## Figures and Tables

**Table 1:** Item-level response distribution for the Subjective Health Status Scale and the Training Satisfaction Scale (*n* = 432).

Item	Strongly Agree		Somewhat Agree		Neither		Somewhat Disagree		Strongly Disagree	
	*n*	%	*n*	%	*n*	%	*n*	%	*n*	%
I feel that I have recovered my energy from the previous day to prepare for training.	117	(27.2)	170	(39.5)	7	(1.6)	96	(22.3)	40	(9.3)
I can get through training to the end without running out of energy.	138	(32.1)	172	(40.0)	70	(16.3)	41	(9.5)	9	(2.1)
I have no gastrointestinal problems such as discomfort, diarrhea, or constipation.	241	(56.0)	78	(18.1)	48	(11.2)	48	(11.2)	15	(3.5)
My bowel movements are regular.	261	(60.7)	90	(20.9)	41	(9.5)	27	(6.3)	11	(2.6)
My skin condition/complexion is good.	219	(50.9)	127	(29.5)	47	(10.9)	31	(7.2)	6	(1.4)
I rarely catch infections such as colds.	188	(43.7)	127	(29.5)	66	(15.3)	34	(7.9)	15	(3.5)
I am able to complete all the training that is planned for the day.	303	(70.5)	89	(20.7)	25	(5.8)	9	(2.1)	4	(0.9)
I can give my full effort.	238	(55.3)	158	(36.7)	21	(4.9)	11	(2.6)	2	(0.5)
I can train with full concentration.	275	(64.0)	121	(28.1)	23	(5.3)	10	(2.3)	1	(0.2)
I can train without feeling anxious.	203	(47.2)	140	(32.6)	61	(14.2)	23	(5.3)	3	(0.7)
I can approach training with a positive attitude.	218	(50.7)	133	(30.9)	53	(12.3)	24	(5.6)	2	(0.5)
I feel refreshed and good.	203	(47.2)	148	(34.4)	52	(12.1)	22	(5.1)	5	(1.2)
I feel that training is enjoyable.	214	(49.8)	133	(30.9)	62	(14.4)	14	(3.3)	7	(1.6)

**Table 2:** Factor loadings for the Subjective Health Status Scale (N = 432). (**a**) Subjective Health Status Scale (maximum likelihood extraction, promax rotation); (**b**) Training Satisfaction Scale (maximum likelihood extraction, promax rotation).

(**a**)		
**Two Factors with Five Items**	**Factor 1**	**Factor 2**
Gastrointestinal/Skin Condition (Cronbach’s α = 0.76)		
My bowel movements are regular.	0.86	−0.03
I have no gastrointestinal problems such as discomfort, diarrhea, or constipation.	0.78	−0.00
My skin condition/complexion is good.	0.45	0.19
Fatigue Recovery/Reduction (Cronbach’s α = 0.76)		
I feel that I have recovered my energy from the previous day to prepare for training.	−0.01	0.85
I can get through training to the end without running out of energy.	−0.01	0.73
I rarely catch infections such as colds. [excluded from final scale]	0.17	0.34
(**b**)		
**One Factor with Seven Items (Cronbach’s α = 0.89)**	**Factor Loadings**	
I feel refreshed and good.	0.86	
I feel that training is enjoyable.	0.79	
I can approach training with a positive attitude.	0.78	
I can train with full concentration.	0.71	
I can train without feeling anxious.	0.69	
I can give my full effort.	0.69	
I am able to complete all the training that is planned for the day.	0.58	

Note: One item (“I rarely catch infections such as colds.”) showed loadings < 0.40 and was removed from the final 5-item subjective health status scale. The two retained factors were labeled “Gastrointestinal/Skin Condition” and “Fatigue Recovery/Reduction.” The correlation between the two factors was r = 0.37. All items loaded on a single factor. Extraction method: maximum likelihood; rotation: promax.

**Table 3:** Descriptive statistics and group comparisons for the Subjective Health Status Scale and Training Satisfaction Scale (N = 432).

			Subjective Health Status						Training Satisfaction ^(3)^		
			Gastrointestinal/ Skin Condition ^(1)^			Fatigue Recovery/ Reduction ^(2)^					
	*n*	(%)	Mean	(SD)	t/F ^(5)^	Mean	(SD)	t/F ^(5)^	Mean	(SD)	t/F ^(5)^
Total	432		12.6	(2.7)		7.7	(1.8)		30.5	(4.6)	
sex											
Men	311	(72)	13.0	(2.3)	2.95 **	7.8	(1.8)	1.35	30.8	(4.5)	2.46 *
Women/Other	121	(28)	11.7	(3.2)		7.5	(1.8)		29.6	(4.8)	
sport categories ^(4)^											
Strength sports	51	(12)	12.2	(3.0)	0.48	8.0	(1.8)	2.00	31.3	(4.5)	2.12
Power sports	115	(27)	12.6	(2.5)		7.4	(1.8)		30.9	(4.8)	
Endurance sports	30	(7)	12.5	(2.5)		8.3	(1.7)		30.3	(5.0)	
Aesthetic/ Weight-class sports	24	(5)	13.0	(3.3)		8.0	(2.2)		32.2	(3.9)	
Team sports	172	(40)	12.7	(2.6)		7.8	(1.8)		30.6	(4.4)	
Other	40	(9)	12.9	(2.4)		7.4	(1.8)		28.9	(5.2)	

(1) Gastrointestinal/Skin condition score: This subscale consisted of three items assessing gastrointestinal symptoms such as discomfort, diarrhea, and constipation (“I do not experience gastrointestinal discomfort, diarrhea, or constipation”), bowel regularity (“My bowel movements are regular”), and skin condition (“My skin condition and complexion are good”). Responses were scored from 5 (best) to 1 (worst), and the total score was calculated by summing the three items (maximum = 15 points, minimum = 3 points). (2) Fatigue Recovery/Reduction score: This subscale consisted of two items: “I feel that I have recovered energy from the previous day in preparation for training,” and “I can complete the training without running out of energy.” Each item was rated from 5 (best) to 1 (worst), and the total score was calculated (maximum = 10 points, minimum = 2 points). (3) Training satisfaction score: This subscale consisted of seven items: “I feel refreshed (pleasant),” “I feel enjoyment during training,” “I can engage positively in training,” “I can concentrate during training,” “I can train without anxiety,” “I exert my full strength,” and “I can complete all the planned training for the day.” Each item was rated from 5 (best) to 1 (worst), and the total score was calculated by summing the seven items (maximum = 35 points, minimum = 7 points). (4) Sport categories: Strength = track and field (jump/sprint); Power = track cycling, swimming, rowing, alpine skiing; Endurance = cross-country skiing, Nordic combined, road cycling; Aesthetic/weight-class = boxing, wrestling; Team = baseball, soccer, soft tennis, water polo; Others = ski jumping, combined events, fencing. (5) Statistical analysis: sex differences were analyzed using *t*-tests, and differences among sport categories were analyzed using one-way ANOVA. t-values are presented for sex comparisons, and F-values for sport category comparisons.* *p* < 0.01, ** *p* < 0.001.

**Table 4:** Correlations among subscales and multiple regression predicting Training Satisfaction.

	1. Gastrointestinal/ Skin Condition	2. Fatigue Recovery/Reduction	3. Training Satisfaction
Inter-scale correlations			
1. Gastrointestinal/Skin Condition	1.00		
2. Fatigue Recovery/Reduction	0.43 *** (0.35~0.51)	1.00	
3. Training Satisfaction	0.44 *** (0.36~0.51)	0.55 *** (0.49~0.62)	1.00

*** *p* < 0.001. Pearson correlation coefficients are shown. Values in parentheses indicate 95% confidence intervals.

**Table 5:** Multiple regression predicting Training Satisfaction.

	Standardized β
Gastrointestinal/Skin Condition	0.24 ***
Fatigue Recovery/Reduction	0.45 ***
Model R^2^ (adjusted R^2^)	0.36 (0.35) ***

*** *p* < 0.001. Dependent variable: Training Satisfaction total score. Predictors: Gastrointestinal/Skin Condition and Fatigue Recovery/Reduction subscale scores. Both predictors were entered simultaneously (forced-entry).

## Data Availability

The datasets generated and/or analyzed during the current study are not publicly available due to restrictions based on participant privacy but are available from the corresponding author on reasonable request.
